# Complete Genome Sequencing and Comparative Phenotypic Analysis Reveal the Discrepancy Between *Clostridioides difficile* ST81 and ST37 Isolates

**DOI:** 10.3389/fmicb.2021.776892

**Published:** 2021-12-21

**Authors:** Tongxuan Su, Wei Chen, Daosheng Wang, Yingchao Cui, Qi Ni, Cen Jiang, Danfeng Dong, Yibing Peng

**Affiliations:** ^1^Department of Laboratory Medicine, Ruijin Hospital, Shanghai Jiao Tong University School of Medicine, Shanghai, China; ^2^Faculty of Medical Laboratory Science, Shanghai Jiao Tong University School of Medicine, Shanghai, China

**Keywords:** *Clostridioides difficile*, ST81, ST37, complete genome sequencing, phenotypic profiling

## Abstract

Toxin A-negative, toxin B-positive *Clostridioides difficile* strains, which primarily include the ST81 and ST37 genotypes, are predominant in *C. difficile* infections leading to antibiotic-associated diarrhea in China. Recently, ST81 has been reported as the most prevalent genotype rather than ST37, although the genetic and functional characteristics of the two genotypes remain ambiguous. In this study, we conducted comprehensive comparative analysis of these two genotypes through complete genome sequencing and phenotypic profiling. The whole genome sequencing revealed that the ST81 and ST37 isolates were closely related genetically with similar gene compositions, and high rate of the core genome shared. The integrative and conjugative elements identified in ST81 were similar to those in ST37, albeit with more diverse and insertion regions. By characterizing the phenotypes related to colonization or survival in the host, we found that the ST81 isolates exhibited robust colonization ability and survival both *in vitro* and *in vivo*, enhanced spore production, and slightly increased motility, which may be attributable to the discrepancy in non-synonymous single-nucleotide polymorphisms in the relevant functional genes. Furthermore, the ST81 isolates displayed a significantly higher rate of resistance to fluoroquinolones compared with the ST37 isolates (94.12% vs. 62.5%) and mostly carried the amino acid substitution Asp426Val in GyrB. In summary, the results of our study indicate that ST81 isolates exhibit enhanced ability to transmit between hosts and survive in harsh environments, providing key genetic insights for further epidemiological investigations and surveillance of *C. difficile* infection.

## Introduction

*Clostridioides difficile* (*C. difficile*) is an anaerobic, spore-forming, and gram-positive pathogen that is a significant cause of healthcare-associated infections around the world (Burke and Lamont, 2014). Its pathogenicity is mainly attributable to two toxins, namely, toxin A and toxin B, which can lead to various symptoms ranging from mild diarrhea to pseudomembranous colitis and even death (Bartlett, 2002). Over the last decade, the morbidity and mortality of *C. difficile* infection have increased globally, making it one of the most common pathogens involved in healthcare-associated infections (Magill et al., 2018).

The multilocus sequence typing (MLST) scheme based on seven housekeeping genes is commonly used to analyze the genetic and phylogenetic features of *C. difficile* (Griffiths et al., 2010) because it has the advantage of allowing interlaboratory comparison. A systematic review in 2016 demonstrated that ST37, a *tcdA*-negative and *tcdB*-positive (A^–^B^+^) strain, was most prevalent in mainland China (Tang et al., 2016). However, several recent studies in Beijing and Shanghai found that the epidemic clone had been replaced by another *tcdA*-negative and *tcdB*-positive strain, ST81 (Qin et al., 2017; Cui et al., 2019; Cheng et al., 2020; Yang et al., 2020), which has caused multiple nosocomial outbreaks. Isolates of *C. difficile* have been classified into five distinct phylogenetic lineages (Janezic and Rupnik, 2015) based on the MLST database, where ST81 and ST37 belong to clade 4 and mainly correspond to ribotype 017 (Wu et al., 2019). In a previous study, Wang (Wang et al., 2018) indicated that ST81 isolates display greater sporulation ability and a high level of fluoroquinolone resistance, which possibly accounts for the prevalence of this strain. Nevertheless, other microbiological characteristics and the genetic features remained unclear. Whole genome sequencing is commonly used to explore genome evolution and the variability of virulence factors (Janezic and Rupnik, 2015). The genomic features and genetic association with virulence have been previously described for ST37 (Liu et al., 2020; Xu et al., 2021), whereas these aspects remain ambiguous for ST81.

In the present study, we conducted a comprehensive comparative analysis between ST81 and ST37 isolates by whole genome sequencing and *in vitro* and *in vivo* phenotypic experiments. In particular, genomic feature analysis was performed with consideration of homologous genes, the PaLoc and CdtLoc regions, integrative and conjugative elements (ICEs), and single-nucleotide polymorphisms (SNPs) of functional genes. To evaluate the potential advantages for persistence and transmission in hosts, the microbiological phenotypes, a *C. difficile* infection mouse model, and the antimicrobial susceptibilities were also explored along with the identification of relevant gene mutations and variations. Overall, it is hoped that our results might provide a robust foundation for the in-depth exploration of functional determinants in *C. difficile* evolution and serve as a reference point for further epidemiological and genomic surveillance of *C. difficile* infection.

## Materials and Methods

### Collection and Identification of *C. difficile* Isolates

Between January 2016 and May 2020, stool samples were collected from inpatients at Ruijin Hospital (Shanghai, China) and subjected to culturing and subsequent identification of *C. difficile* as described previously (Dong et al., 2013). Isolates were stored in a Cryobank (Mast Group Ltd., Bootle, United Kingdom) at −80°C. Toxin genes *tcdA* and *tcdB* as well as binary toxin genes *cdtA* and *cdtB* were amplified. MLST was performed using the method established in 2010 (Griffiths et al., 2010). Sequences were submitted to^1^ to obtain the allele profile and determine the sequence type (ST).

### Whole Genome Sequencing, Assembly, and Annotation

Isolates were anaerobically cultured on BHIS [brain heart infusion (Oxoid Ltd.) supplemented with 5 g/L yeast extract (Oxoid Ltd.) and 0.1% L-cysteine (Sangon Biotech)] plates at 37°C for 48 h. Single clones were picked and re-cultured in BHI (brain heart infusion) broth for 18 h followed by genomic DNA extraction. The genomic DNA was extracted using the Wizard Genomic DNA Purification Kit (Promega) and quantified on a TBS-380 fluorometer (Turner BioSystems Inc., Sunnyvale, CA, United States). To obtain high-quality DNA, it was required that the OD_260_/OD_280_ ratio was between 1.8 and 2.0 and the amount was greater than 20 μg. Draft genome sequencing was performed on the Illumina HiSeq platform, while complete genomes were sequenced on PacBio RS II Single Molecule Real Time and Illumina sequencing platforms at Shanghai Majorbio Bio-pharm Technology Co., Ltd. (Shanghai, China). Raw reads with adapter contamination, 5′ ambiguous bases, low quality, 10% ambiguous bases, or fewer than 25 bp were discarded. The clean data were assembled using SOAPdenovo2 for draft genomes and Canu or SPAdes v.3.8.0 for complete genomes. Prediction of the coding sequences (CDSs), tRNA, and rRNA was carried out using Glimmer v3.02 (Delcher et al., 2007), tRNAscan-SE v2.0, and Barrnap (Borodovsky and McIninch, 1993), respectively. Sequence alignment tools such as BLAST, Diamond, and HMMER were used for annotation of the predicted CDSs. Each set of query proteins was aligned with the following databases: Non-Redundant Protein Database (NR), Swiss-Prot, Pfam, Gene Ontology (GO), Clusters of Orthologous Groups (COG), and Kyoto Encyclopedia of Genes and Genomes (KEGG). Prophages and gene islands were identified using Phage_Finder (Fouts, 2006) and IslandViewer 4 (Bertelli et al., 2017), respectively. The circular genomes were constructed using the Circos software based on the predicted ORFs, rRNA, tRNA, prophages, gene islands, and GC skew information. Online tool OrthoMCL^2^ in the Majorbio Cloud Platform^3^ were used for comparative analysis to determine the shared and unique genes harbored by different isolates.

### Determination of SNPs and Phylogenetic Analysis

Single-nucleotide polymorphisms were detected using the BWA software^4^ with the complete genome of CD630 (accession number AM180355.1) as a reference. Reads generated by PCR duplication were removed with the Picard Tools software and the reads were then realigned in the GATK software to eliminate the false positive SNPs. After filtration of the sites with low sequence depth and comparison quality values using the Snippy 4.6.0 software, the SNPs were annotated to determine their potential effects on annotated genes using SnpEff^5^. Phylogenetic analysis was conducted using the MEGA 6.0 software. The phylogenetic tree was graphically constructed based on concatenated alignments of SNPs or single copy ortholog sequences identified by OrthoFinder 2.3.3^6^.

### Sequence Analysis of the PaLoc and CdtLoc Regions

The PaLoc and CdtLoc regions were determined by comparison with the genome of the reference strain CD630. Orthologous genes with over 80% coverage and 90% identity were detected by BLAST searching. Analysis of the SNPs and indels was also conducted by the method of SNPs described above.

### Detection of Mobile Genetic Elements (MGEs)

Mobile Genetic Elements including integrative and conjugative elements (ICEs) of *C. difficile* were searched for in the ICEberg platform^7^ with parameters of 0.0001 Expect threshold and 15 Word Length by gapped alignments and were further identified in BLAST. ICEs were defined as >80% nucleotide identity and coverage with the referenced sequences.

### *C. difficile* Infection Mouse Model

To assess the *in vivo* colonization and survival ability of the clinical isolates, a *C. difficile* infection mouse model was established as described previously with slight modifications (Chen et al., 2008). Briefly, 6-week-old C57BL/6 mice were treated with drinking water containing an antibiotic cocktail composed of 0.4 mg/mL kanamycin, 0.035 mg/mL gentamicin, 0.042 mg/mL colistin, 0.215 mg/mL metronidazole, and 0.045 mg/mL vancomycin for 3 days, followed by regular water for 2 days and intraperitoneal injection of 20 mg/kg clindamycin 1 day prior to infection. On the day of infection, the mice were infected with 10^6^ or 10^6^CFU each (competitive assays) spores through oral gavage. Stool samples were collected for *C. difficile* cultures each day from day 0 (prior infection) to day 3 (post infection). For competitive assays, samples were cultured on plates with and without addition of 64 mg/L moxifloxacin to discriminate ST81 isolates from the total bacterial cells. Colonies were counted and the CFUs per milligram of feces were calculated.

### Investigation of Phenotypic Features *In vitro*

Freshly prepared *C. difficile* isolates were cultured in BHIS broth overnight at 37°C for subsequent phenotypic experiments. For sporulation capacity determination, the bacterial cultures were adjusted to OD_600_ = 0.6. The cells were washed twice, incubated at 60°C for 25 min, diluted in BHIS broth at dilutions of 1:10^3^, 1:10^4^, and 1:10^5^, and plated on BHIS plates supplemented with 0.1% sodium taurocholate (Sangon Biotech). Colonies were counted and recorded at the number of 30–300 per plate after 48 h of anaerobic incubation. Biofilm formation was measured as described previously with slight modifications (Dong et al., 2014b). Briefly, 20 μL aliquots of overnight cultures (OD_600_ = 1.0) were seeded into 24-well plates with 1 mL of BHIS broth. After incubation for 24 h and removal of the supernatant, the wells were gently washed twice with PBS then air dried for 10 min at room temperature. Biomass was measured by adding 1% crystal violet (Sangon Biotech) and decolorization with methanol. The extracted dye was diluted at tenfold and detected at 570 nm *via* a spectrophotometer (Thermo Scientific Multiskan GO). The absorbance was normalized to that of CD630. Motility tests were conducted by stabbing 2 μL aliquots of bacterial cultures (OD_600_ = 0.5) into semi-solid BHI agar plates containing 0.3% agar (Edwards et al., 2016). The swimming diameter was measured after 48 h of anaerobic incubation.

To evaluate the interaction between bacterial and host cells, we performed *C. difficile* adherence and intracellular survival assays. Human epithelial Caco-2 cells (ATCC HTB-37) and human monocyte THP-1 cells (ATCC TIB-202) were purchased from ATCC and cultured in Dulbecco’s modified Eagle’s medium (DMEM) and RPMI1640 medium, respectively, supplemented with 10% fetal bovine serum and 50 mg/L penicillin/streptomycin. *C. difficile* isolates were freshly prepared in BHIS broth and adjusted to OD_600_ = 1.0. For the adherence assay, 100 μL aliquots of bacterial culture were harvested, washed, resuspended in DMEM medium, and added to each well of Caco-2 cells, which had been pre-cultured for 48 h. After incubation at multiplicity of infection (MOI) 10:1 for 90 min, the Caco-2 cells were washed three times to remove unadhered bacteria, detached with PBS containing 1% (w/v) Triton X-100 for 5 min, and plated on BHIS plates to measure the number of adherent bacterial cells. For the intracellular survival assay, THP-1 cells were seeded onto 24-well plates (5 × 10^5^ per well) and pretreated with 20 ng/mL of phorbol 12-myristate 13-acetate (Sigma Aldrich) overnight. Cells were then cultured with fresh medium without PMA and incubated with the *C. difficile* isolates at MOI 10:1 for 5 h. After removal of supernatants, cells were treated with 5 mg/L metronidazole for an hour to kill extracellular bacterial cells and washed with PBS twice, further lysed with PBS containing 0.1% (w/v) Triton X-100 to release intracellular live bacterial cells. The lysates were centrifuged at 3000 rpm for 5 min then pellets were resuspended and plated on BHIS plates for CFU counting at appropriate dilutions. All the phenotypic experiments were performed at least three times using CD630 as an internal control.

### Antimicrobial Susceptibility Testing and Detection and Sequencing of Resistance Genes

Antimicrobial susceptibility testing was performed by the agar dilution method according to Clinical and Laboratory Standards Institute guidelines with *Bacteroides fragilis* ATCC 25289 and *C. difficile* ATCC 700057 as quality controls (CLSI, 2011). Eight antibiotics, including penicillin, rifampicin, clindamycin, tetracycline, cefoxitin, moxifloxacin, metronidazole, and vancomycin, were selected to determine the minimum inhibition concentrations (MICs) for *C. difficile* (Dong et al., 2013).

The gyrase subunit genes *gyrA* and *gyrB* along with the gene encoding the β-subunit of RNA polymerase *rpoB*, which are responsible for fluoroquinolone and rifampicin resistance, respectively, were amplified and sequenced as described previously (Huang et al., 2009). Sequences were aligned to CD630 (GenBank accession no. AM180355.1) using the BLAST software.

### Statistical Analysis

All statistical analyses were performed using SPSS version 16.0. The Mann–Whitney test was used to analyze the adhesion, macrophage survival, and sporulation assay data, while the Welch’s *t*-test was used for the biofilm formation and motility data. The chi-square test and log rank test were used to evaluate the resistance rates and survivorship curves of the strains in the presence of antibiotics. The data visualizations and graphs were produced using GraphPad Prism version 8.0 and R software version 4.0.5. The heatmaps and schematic diagrams of the gene elements were generated using the pheatmap and gggenes package in the R software.

## Results

### Phylogenetic Analysis and Genomic Features of Selected ST81 and ST37 Isolates

**FIGURE 1:**
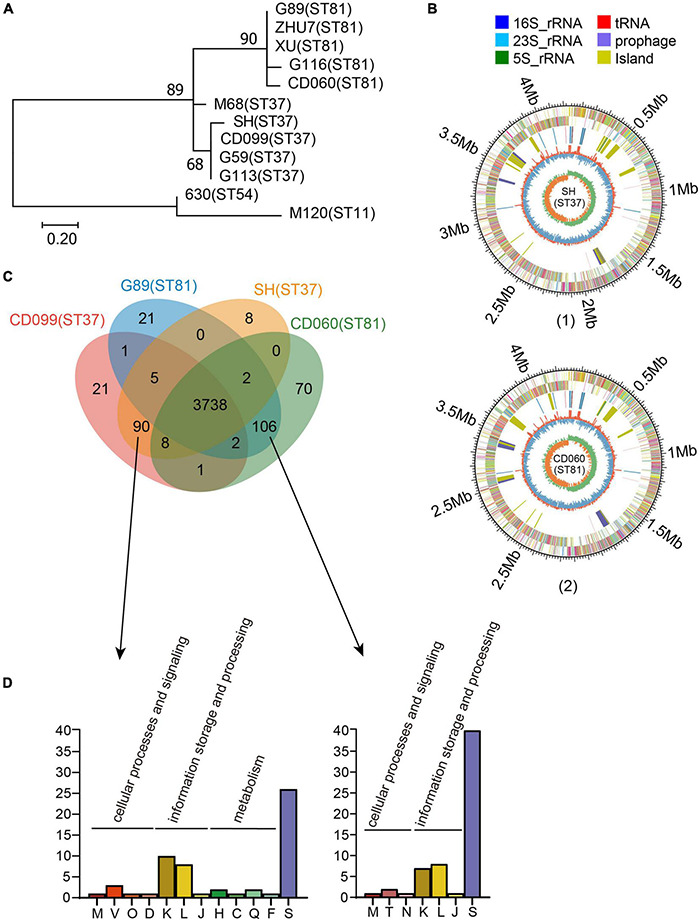
Phylogenetic analysis and genomic features of the ST81 and ST37 isolates. **(A)** Analysis of phylogenetic tree depicting the relationships of *C. difficile* isolates based on SNPs by draft genome sequencing. CD630, CDM68, and CDM120 were used as reference strains. The sequence types are labeled in parentheses. **(B)** Schematic diagram of the complete genomes of *C. difficile* for isolate SH as a representative of ST37 (1) and for isolate CD060 as a representative of ST81 (2). The circle diagram indicated as follows (from the outer layer inward): the genome size, the annotated COG genes on the forward strand, the annotated COG genes on the reverse strand, 16S rRNA (dark blue), 23S rRNA (pale blue), 5S rRNA (green), tRNA (red), prophage (purple), island (yellow), GC content, and GC skew (G−C/G+C). **(C)** Venn diagram showing the number of shared and unique genes for the ST81 and ST37 isolates. **(D)** Enrichment of COG annotation of unique genes in ST37 (left) and ST81 (right). The classes are as follows: [M] cell wall/membrane/envelope biogenesis; [V] defense mechanisms; [O] post-translational modification, protein turnover, chaperones; [D] cell cycle control, cell division, chromosome partitioning; [K] transcription; [L] replication, recombination, and repair; [J] translation, ribosomal structure, and biogenesis; [H] coenzyme transport and metabolism; [C] energy production and conversion; [Q] secondary metabolites biosynthesis, transport, and catabolism; [F] nucleotide transport and metabolism; [S] function unknown; [T] signal transduction mechanisms; [N] cell motility.

In total, 97 *tcdA*^–^*tcdB*^+^ isolates were collected during the study period, among which 56 (57.73%) and 18 (18.56%) were genotyped as ST81 and ST37, respectively. Five isolates of ST81 and 4 isolates of ST37 were randomly selected for whole genome sequencing. These nine sequences in addition to three annotated genomes, including CD630 (ST54, A^+^B^+^), CDM120 (ST11, A^+^B^+^), and CDM68 (ST37, A^–^B^+^), were analyzed in the MEGA 7.0 software to investigate the phylogeny based on SNPs. The phylogenetic tree clearly categorized these isolates into two groups (Figure 1A). Genomes of isolates belonging to the same sequence type were tightly clustered together. The reference strain CDM68, verified as ST37, was more closely related to the ST37 group with high bootstrap values. In addition, single copy ortholog sequences were also screened and used to evaluate phylogenetic analysis, which displayed a high similarity of ortholog sequences between ST81 and ST37 isolates (Supplementary Figure 1). Overall, the ST81 and ST37 isolates were found to be closely related.

To obtain a better understanding of the genetic features and genomic differences between isolates of ST81 and ST37, we selected four of them, namely, CD060 (ST81), G89 (ST81), CD099 (ST37), and SH (ST37), for complete genome sequencing. The genomic features of these isolates are summarized in Supplementary Table 1. The selected isolates were similar in terms of genome size (4.29 to 4.34 Mb), GC content (28.76 to 28.80%) and number of coding sequences (3898 to 4009) and tRNA and rRNA sequences. Schematic diagrams of the complete chromosomal genomes and plasmid genomes are presented in Figure 1B and Supplementary Figure 2. Only one plasmid was identified in each isolate, which shared 100% coverage and identity with that in strain CDM68.

### Comparative Genomic Analysis Between ST37 and ST81 Isolates

As shown in Supplementary Figure 3, the four isolates harbored similar gene compositions in functional categories based on the COG database. Approximately 86% (85.73 to 86.99%) of the CDSs could be specifically assigned to clusters of the COG family comprising 21 categories, among which function unknown (Class S, 996 to 1046 genes) occupied the largest proportion followed by transcription (Class K, 334 to 338 genes), amino acid transport and metabolism (Class E, 280 to 282 genes), carbohydrate transport and metabolism (Class G, 265 to 266 genes), signal transduction mechanisms (Class T, 192 to 195 genes), and so on.

We then analyzed the shared and unique genes between isolates of ST81 and ST37 (Figure 1C). Core genomes are usually used to evaluate the genomic diversity within species. In total, 3738 CDSs in the core genome were shared by all isolates, suggesting a close relationship between these isolates. On the other hand, there were 90 and 109 unique genes harbored by the ST81 and ST37 isolates, among which 51 and 60 genes could be assigned to COG functional categories (Figure 1D), respectively. The largest proportion of unique genes belonged to function unknown (Class S), followed by transcription (Class K) and replication, recombination, and repair (Class L). It is noteworthy that 13.33% (12/90) of the unique genes in the ST37 isolates were enriched in in cellular processes and signaling (Class M, Class V, Class O, and Class D) and metabolism (Class H, Class C, Class Q, and Class F), much more than 3.77% (4/106) in ST81 isolates (Figure 1D), which may endow these isolates with the ability to defend against adverse stimuli and use extra energy to better adapt to the environment. For the ST81 isolates, more genes of unknown function were found, indicating that further efforts are needed to determine the putative functions of genes in these isolates.

### Analysis of the ICEs in the ST81 and ST37 Isolates

**FIGURE 2:**
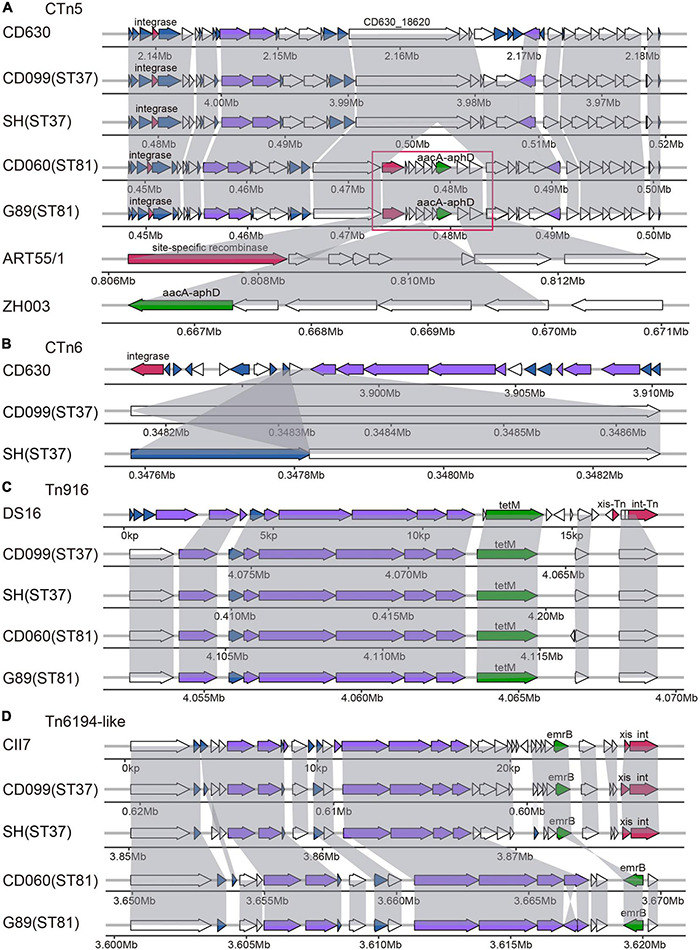
Schematic diagram of representative ICEs in selected ST81 and ST37 isolates as well as reference strains, including CTn*5* **(A)**, CTn*6* **(B)**, Tn*916* **(C)**, and Tn*6194*-like **(D)**. ORFs involved in similar function were showed in the same color: blue, conjugative transposon protein; red, excisionase and integrase; purple, conjugation module; green, antibiotic resistant gene; white, other functions. The large insertion region in the CTn*5* is indicated by the red frame with the ORFs homologous to *Coprococcus* sp. ART55/1 and to *Campylobacter jejuni* strain ZH003.

In total, nine putative ICEs could be annotated to the genomes of these isolates by the ICEberg platform. As listed in Supplementary Table 2, the genetic structures of the elements were almost identical within STs. However, only two elements (CTn*5* and Tn*6194*-like) in ST37 isolates and three (CTn*2*, CTn*5*, and Tn*6103*) in ST81 isolates contained both putative conjugative machinery and integrase responsible for excision and integration, which were considered essential for ICEs. Besides, distinct ICEs could be referred to the same genome regions, probably due to the homology of ICEs in *C. difficile* (Sebaihia et al., 2006). CTn*1* was homologous with CTn*7* and Tn*6194*-like, CTn*2* with CTn*5* and Tn*6103*, and Tn*5397* with Tn*916*. Thus, we chose those with the most matched ORFs as representatives for further exploration in the genome sequences of the elements, including CTn*5*, CTn*6*, Tn*916*, and Tn*6194*-like (Figure 2).

Specifically, putative ICE of CTn*5* was identified in the four isolates, sharing 31 to 32 matched ORFs with that found in CD630 with 38 ORFs. Compared to the ST37 isolates, the ST81 isolates had a 10.4 kb insertion fragment in gene CD630_18620, encoding a putative DNA/RNA helicase. The large insertion region was composed of nine genes. BLAST searching results revealed that the first and last two genes displayed approximately 98 to 100% coverage and 90.3 to 94% identity with those in *Coprococcus* sp. ART55/1, mainly encoding site-specific recombinase and plasmid recombination enzymes. Meanwhile, the middle of the insertion region could be aligned to genes in *Campylobacter jejuni* strain ZH003 with 94.0 to 100% identity, including the *aacA-aphD* gene, which plays important roles in resistance to gentamicin, tobramycin, and kanamycin. As for CTn*6*, it contained an integrase in reference strain belonging to the lambdoid phage family and only presented one to two ORFs in the ST37 isolates. Tn*916* was first reported in *Enterococcus faecalis* DS16, carrying the tetracycline resistance gene *tet*(M) and the indicated marker gene *intTn*. In this study, the Tn*916* transposons were very similar in the ST81 and ST37 isolates with slight variations compared to the reference gene structure. The upper regions were replaced by a gene encoding an FtsK/SpoIIIE family protein, and the downstream genes *xis-Tn* and *int-Tn* encoded excisionase and integrase was lost. Although the *tet*(M) leader peptide was lost, the resistance gene was retained. Another putative ICE characterized was the Tn*6194*-like element, carrying *erm*B gene and referenced to *C. difficile* CII7. This element in the ST37 isolates displayed higher similarity to the standard schematic diagram and still contained genes *xis* and *int* responsible for its excision and integration. However, in the ST81 isolates, this element was devoid of integrase and more than half of the ORFs with several insertion fragments identified, and the macrolide–lincosamide–streptogramin B (MLS_*B*_) resistance gene *erm*B was oriented in the reverse direction. Overall, the ICEs in the ST81 isolates appeared more heterogeneous with respect to the standard gene composition of the elements. Interestingly, approximately 52.63% (10/19) of the insertion genes found in the elements were unique genes for the ST81 isolates. The genetic features of the elements suggested that the distinct genomes between the ST37 and ST81 isolates may be partially attributable to horizontal gene transfer by ICEs.

### General Features of the PaLoc and CdtLoc Regions in the ST81 and ST37 Isolates

All four of the *C. difficile* isolates, which were identified as *tcdA*^–^*tcdB*^+^*cdtA*^–^*cdtB*^–^, had an incomplete and discontinuous *tcdA* gene in the PaLoc region and the absence of the *cdtR*, *cdtA*, and *cdtB* genes in the CdtLoc region (Supplementary Figure 4). Specifically, a gene annotated as a hypothetical protein was found to be inserted upstream of the *cdd2* gene. A gained stop codon and four frameshift mutations in the *tcdA* gene were found in these isolates, leading to the termination of *tcdA* gene translation. The number and distribution of SNPs in *tcdR*, *tcdB*, *tcdE*, *tcdA*, and *tcdC* were totally identical, except that the ST81 isolates had an additional missense variant in the *tcdA* gene. Overall, no obvious differences in the PaLoc and CdtLoc regions were found between the ST81 and ST37 isolates.

### Functional Analysis of SNPs in Variant Genes Among the ST81 and ST37 Isolates

**FIGURE 3:**
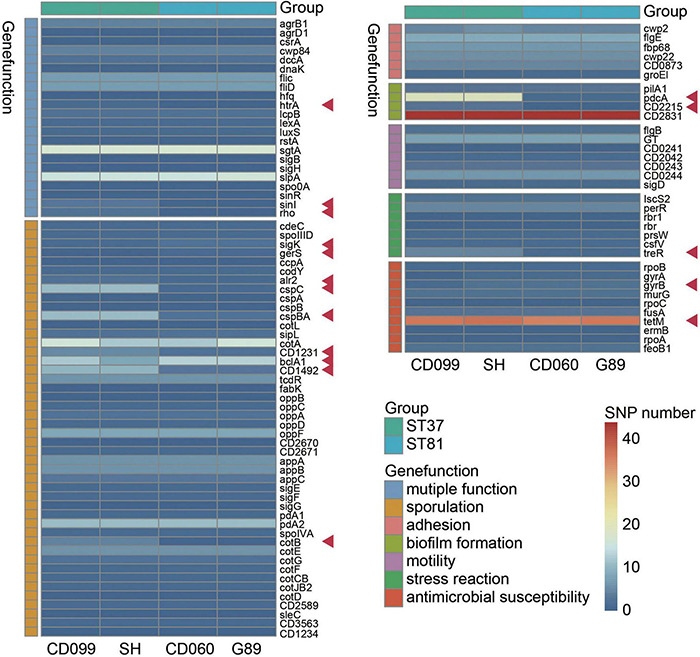
Functional analysis of non-synonymous SNPs in variant genes among the ST81 and ST37 isolates. Genes clustered in the same color refer to the same gene function indicated aside. The ST81 and ST37 isolates are labeled below and marked in light blue and light green, respectively. The colors from blue to red in the middle represent the number of SNPs identified. Genes with different numbers of SNPs between STs are indicated by red arrows.

To further determine whether variant genes related to potential phenotypes differed between the ST81 and ST37 isolates, we next explored the SNPs in genes that may be associated with colonization or survival in the host. After alignment to CD630, a total of 46109 to 51517 SNPs were detected, among which 11121 to 12305 were non-synonymous. Fewer SNPs, in terms of both total and non-synonymous SNPs, were detected in the ST81 isolates (Supplementary Table 3). Seven kinds of key functional genes were categorized based on current reports, including sporulation, biofilm formation, motility, adhesion, stress reaction, antimicrobial susceptibility, and multiple functions (Figure 3). Synonymous variants and mutations in the intergenic region were discarded. Supplementary Table 4 presents the detailed variant sites in the relevant genes.

As expected, the ST81 isolates usually harbored fewer non-synonymous SNPs in the relevant functional genes. Most differential SNPs were identified in genes related to sporulation, including *sigK*, *gerS*, *alr2*, *cspC*, *cspBA*, *CD1231*, *CD1492*, and *cotB*. One of the most noticeable differences was detected in two spore germination genes, *cspC* (Rohlfing et al., 2019) and *cspBA* (Kevorkian and Shen, 2017). There were eleven SNPs in each gene for the ST37 isolates but only one or two for the ST81 isolates. In addition, genes *CD1231* (Serrano et al., 2016) and *CD1492* (Childress et al., 2016), which may play a role in the regulation of spore morphogenesis and sporulation, also exhibited less polymorphism in the ST81 isolates. Meanwhile, genes such as *sigK* (Serrano et al., 2016), *gerS* (Diaz et al., 2018), and *bclA1* (Díaz-González et al., 2015) involved in sporulation displayed a slightly larger number of SNPs in the ST81 isolates. In the case of *pcdA*, which encodes the phosphodiesterase for c-di-GMP in *C. difficile* and has been linked to increased biofilm formation upon mutation (Purcell et al., 2017), one SNP was detected in the ST81 isolates compared with 19 SNPs in the ST37 isolates. Similar results were obtained for *CD2215*, a SinR-like regulator participating in biofilm formation (Poquet et al., 2018). Concerning the stress reaction, the gene *treR*, which is critical for the tolerance of oxidative stress in *Streptococcus mutans* (Baker et al., 2018), also exhibited high polymorphism in the ST37 isolates with four missense mutations and a frameshift variant. Meanwhile, larger numbers of SNPs were also detected in multiple functional genes such as *htrA* (Bakker et al., 2014), *sinI* (Ciftci et al., 2019), and *rho* (Trzilova et al., 2020), which play essential roles in various processes such as sporulation, adhesion, and motility. No other differences were found in the gene sets associated with motility or cell adhesion. Another striking finding is that the ST81 isolates carried an extra Asp426 mutation in *gyrB*, which is strongly associated with acquired resistance to fourth-generation fluoroquinolones. Altogether, our findings indicated that the ST81 and ST37 isolates had discrepant non-synonymous SNPs in multiple functional genes, leading us to an in-depth experimental exploration of the related phenotypes.

### Phenotypic Profiling of the ST81 and ST37 Isolates

**FIGURE 4:**
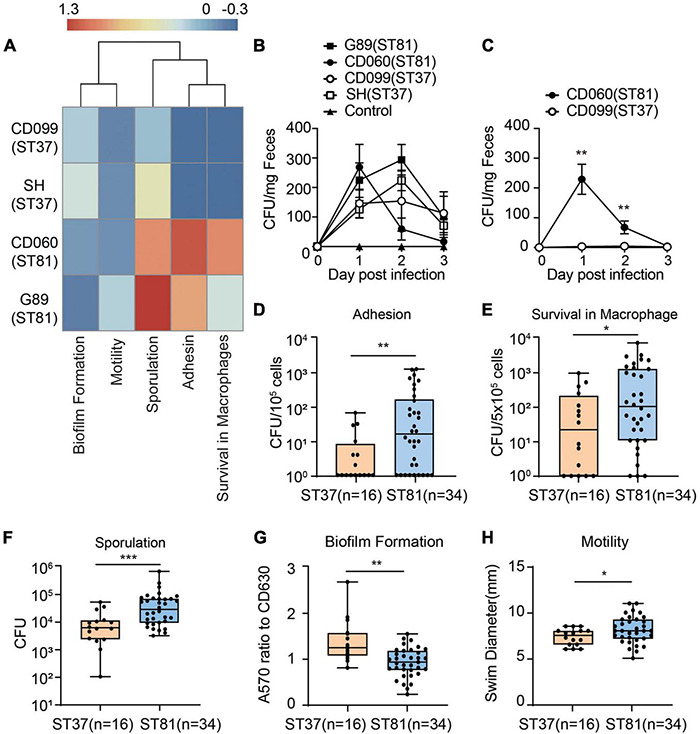
Phenotypic characterization of the ST81 and ST37 isolates. **(A)** Phenotypic profiles of the four sequenced isolates compared to CD630. Results of different phenotypes were adjusted as follows: adhesion, survival from macrophage and sporulation data were calculated as logCFU (isolate)-logCFU (CD630); biofilm and motility data were calculated as a ratio (isolate-CD630)/CD630. The heatmap was generated by setting enhanced phenotype (>0) and decreased phenotype (<0). The colors from blue to red represent the degree of difference. **(B,C)** Stool samples from mice infected with various isolates **(B)** or competitive assays **(C)** were collected from day 0 to day 3 post infection and subjected to *C. difficile* culturing. Colonies were counted and quantified per milligram of fecal samples. **(D–H)** Colony counts of clinical *C. difficile* isolates adhered to Caco-2 cells **(D)**, survival in THP-1 cells **(E)**, and spore production **(F)**. **(G)** Biomass was measured by the absorbance at 570 nm and normalized to CD630. **(H)** Swim diameters were recorded to assess the motility. Data are shown as min to max. All experiments were repeated at least three times independently. Significant differences are indicated with *p < 0.05, **p < 0.01,***p < 0.001.

Although the phylogenetic analysis revealed a close relationship between the two groups, the discrepancy with respect to the SNPs in functional genes motivated us to further investigate the phenotypic diversity of the ST81 and ST37 isolates. First, we used the four isolates subjected to whole genome sequencing to determine the virulence- or persistence-related phenotypes by using CD630 as a reference. The heatmap in Figure 4A clearly illustrated that the two ST81 isolates displayed stronger sporulation, robust ability of epithelial cell adhesin, and enhanced survival ability in macrophages but reduced biofilm formation. The distinct phenotype of sporulation and survival ability in macrophages may be attributable to the non-synonymous SNPs because the ST81 isolates usually carried fewer missense mutations in genes encoding the respective functional proteins. Meanwhile, the increased biofilm formation ability of the ST37 isolates presumably resulted from the greater number of missense variants in *pdcA*. In addition, the phenotypic features of motility and cell adhesion may be attributable to the different degrees of polymorphism in the gene sets with multiple functions including *htrA*, *sinI*, and *rho*.

Furthermore, we utilized a *C. difficile* infection mouse model to compare the colonization and survival ability of the ST81 and ST37 isolates. Mice were infected with the four strains and feces were collected at day 0 to day 3 to evaluated burdens of each isolate. The results revealed that no significant differences of CFUs counted were found between selected ST81 and ST37 isolates (Figure 4B). As for the competitive assay, mice were infected with a cell mixture with equal amount of isolate CD099 (ST37) and CD060 (ST81). Due to the higher antimicrobial susceptibility to moxifloxacin, we assessed burden of CD060 (ST81) by counting CFUs on BHIS plates with 64 mg/L moxifloxacin, and taking the CFU numbers on BHIS plates as the total *C. difficile* cells. As illustrated in Figure 4C, CD060 (ST81) accounted for a remarkably larger amount on day 1 and day 2, suggesting that ST81 isolates might be more competitive for colonization *in vivo* than ST37. The failure for CD099 (ST37) to colonize in the presence of CD060 (ST81) might be attributed to competition for binding sites or nutrition in the hash intestinal environment or other underlying mechanisms, which need to be further investigated. In general, the results of infection model further confirmed the phenotypic features that ST81 isolates might exhibit enhanced advantages of persistence and colonization *in vivo*.

Next, we used the collected clinical isolates (Figures 4D–H), namely, 16 isolates for ST37 and 34 isolates for ST81, to verify the phenotypic features described previously. In accordance with the results from the four isolates, the group of ST81 isolates produced significantly more spores (mean 6.8 × 10^5^ vs. 1.2 × 10^5^ CFU), exhibited remarkably enhanced adhesion ability (mean 2.0 × 10^2^ vs. 1.0 × 10^1^ CFU per 10^5^ cells), displayed improved survival in macrophages (mean 6.5 × 10^2^ vs. 1.5 × 10^2^ CFU per 5 × 10^5^ cells), and showed slightly increased motility (mean 8.12 vs. 7.33 mm). In addition, the ST81 isolates generated less biomass as measured by the absorbance at 570 nm than the ST37 isolates (0.92 vs. 1.37 with respect to CD630). Thus, the phenotypic profiling results indicated that the ST81 isolates may possess advantages with respect to adaptation and dissemination in both the host and environment.

### Antimicrobial Susceptibility and Resistance Gene Analysis for the ST81 and ST37 Isolates

**FIGURE 5:**
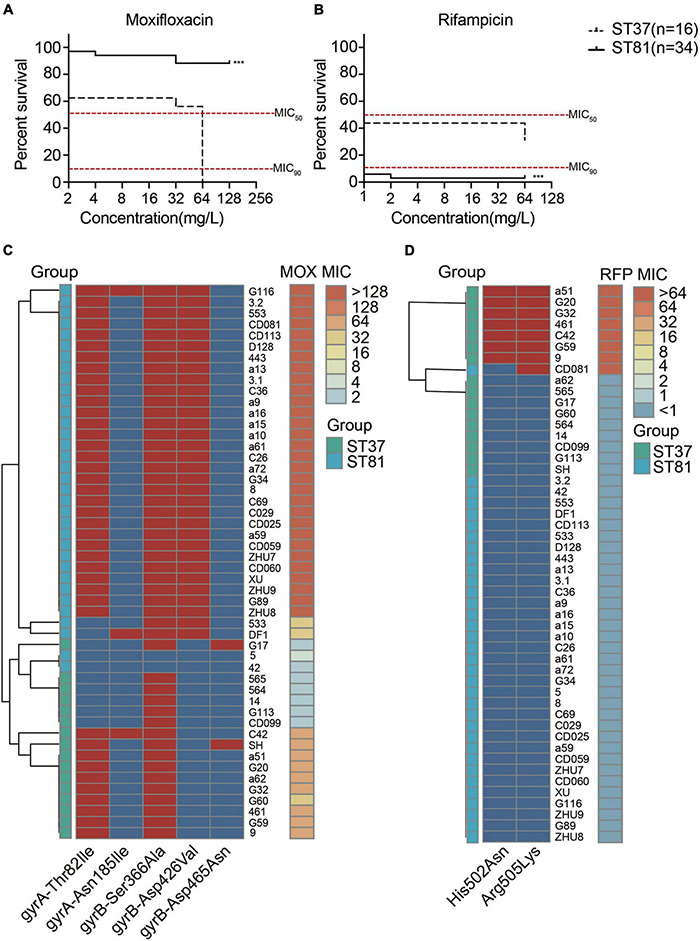
Antimicrobial susceptibility and amino acid substitutions in the moxifloxacin and rifampicin resistance genes. **(A,B)** Survival curves of isolates under various concentrations of moxifloxacin **(A)** and rifampicin **(B)**. MIC_50_ and MIC_90_ are indicated with red lines. The ST81 and ST37 isolates are indicated with solid and dashed lines, respectively. Significant differences are indicated with ***p < 0.001. **(C,D)** Mutations of resistance genes associated with the MIC values toward moxifloxacin **(C)** and rifampicin **(D)** for each of the clinical isolates. The MIC values are shown on the right vertical axes and marked in colors from light blue to orange. The presence (red) and absence (blue) of mutations indicated on the horizontal axes were in the middle part. Isolates genotyped as ST81 and ST37 are indicated in light blue and light green, respectively. MOX, moxifloxacin. RFP, rifampicin.

All of the isolates were subjected to antimicrobial susceptibility tests and the results are presented in Supplementary Table 5. Generally, all of the isolates were susceptible to metronidazole and vancomycin but resistant to clindamycin and cefoxitin. No significant difference in resistance to tetracycline was observed for either of the two groups. The moxifloxacin resistance of the ST81 isolates reached 94.12%, compared with 62.50% for the ST37 isolates. However, the frequency of resistance to rifampicin was 2.94 and 43.75% for the ST81 and ST37 isolates, respectively. In accordance with these results, the percentage survival curves further verified the enhanced resistance to moxifloxacin for the ST81 isolates and to rifampicin for the ST37 isolates, as did the MIC_90_ and MIC_50_ values (Figures 5A,B). Fluoroquinolone and rifampicin resistance is considered closely related to mutations in the *gyrA*, *gyrB*, and *rpoB* genes. Thus, we amplified and sequenced the full lengths of these genes for all of the isolates and mapped the MIC values and point mutations for each isolate to the phylogeny. As shown in Figure 5C, the mutation of Thr82 to Ile in GyrA and Asp426 to Val in GyrB was significantly associated with the MIC values toward moxifloxacin. Isolates carrying either mutation exhibited some resistance to moxifloxacin, while those bearing both mutations displayed MIC values of up to >128 mg/L. Significantly, approximately 88.2% of the ST81 isolates carried both mutations, exhibiting increased MIC values and high resistance toward moxifloxacin. Furthermore, we found that the MIC-based phylogenetic tree was consistent with the MLST-based clustering.

As for the rifampicin resistance, two mutations in RpoB, namely, His502 to Asn and Arg505 to Lys, were identified. The presence of either mutation in the isolates afforded resistance to the antibiotic. The heatmap also illustrated the relationship between mutations in *rpoB* and the MIC values toward rifampicin (Figure 5D). Almost all of the rifampicin-resistant isolates, with the exception of CD081, belonged to ST37.

## Discussion

*Clostridioides difficile* infection is considered one of the most urgent threats to public health worldwide. In Asia, the *tcdA*^–^*tcdB*^+^ *C. difficile* strain ribotype 017/ST37 was previously reported to be the most epidemic clone (Tang et al., 2016; Imwattana et al., 2019). Recently, however, numerous studies have demonstrated that another *tcdA*^–^*tcdB*^+^ strain, ST81, has emerged as the predominant clone in northern and eastern China (Qin et al., 2017; Cui et al., 2019; Cheng et al., 2020; Yang et al., 2020). In accordance with this, in our follow-up monitoring, the prevalence of ST81 was found to be remarkably higher than that of ST37 compared with our previous findings at the same institution in Shanghai (Dong et al., 2014b). Genomes of *C. difficile* typically exhibit high levels of diversity and plasticity with low levels of gene conservation (Knight et al., 2015). The distribution of *C. difficile* ST37 was determined to be region dependent with high sequence diversification based on a large-scale genomic investigation in China (Xu et al., 2021). Herein, isolates belonging to the two sequence types were found to be tightly clustered, possibly owing to a common origin. Genotypes ST81 and ST37 belong to the same cluster clade of phylogenetic lineage in the MLST database with only one allelic *atpA* variant (Kuwata et al., 2015), and they displayed a very close relationship in our study according to phylogenetic analysis with a high rate of shared core genes. Owing to the limited sequencing information and phenotypic characterization of ST81 isolates available, the discrepancy between these two genotypes required further elucidation.

As essential mobile genetic elements, ICEs are responsible for horizontal gene transfer, driving increased genetic diversity and the acquisition of exogenous genes (He et al., 2010). Transposons, including Tn*916*, transposons from the Tn*916*-like family, Tn*5397*, Tn*5397*-like elements, Tn*6002*-like elements, and so on, have been found to be widely dispersed in ST37 isolates (Xu et al., 2021). Herein, nine types of ICEs could be annotated to the genome of these isolates and we selected four as representatives, including, CTn*5*, CTn*6*, Tn*916*, and Tn*6194*-like, on the basis of the most matched ORFs. With the exception of CTn*6*, ST81 had all of the transposons found in ST37 but with more insertions and deletions compared with the reference sequences. Tn*916*, a well-known *tet*(M)-carrying element, is considered to play a key role in *tet*(M) gene acquisition and tetracycline resistance (Xu et al., 2021). Thus, the ST81 and ST37 isolates displayed similar rates of resistance to tetracycline. This element in both the ST81 and ST37 isolates contained a gene encoding an FtsK/SpoIIIE family protein instead of the upper region, as has been previously described for ST37 (Dong et al., 2014a). Our previous study suggested that this replacement could afford decreased conjugative ability, leading to the dissemination of the element caused by clonal spread in ST37 isolates. The presence of this element in the ST81 isolates further suggested their clonal relatedness and indicated that they are derived from the same sublineages. The sequences of the determinants in *C. difficile* commonly exhibit high similarity with those in other pathogens, which is associated with the development of antibiotic resistance. Herein, a large insertion fragment in CTn*5* was found in the ST81 isolates, comprising of recombinase homogenous with that from *Coprococcus* spp. and *aacA-aphD* gene from *C. jejuni* ZH003. The *aacA-aphD* gene is known as an aminoglycoside resistance determinant, providing resistance to gentamicin, tobramycin, and kanamycin (Rouch et al., 1987). Because the clinical use of aminoglycosides is reported to be significantly associated with *C. difficile* infection (Chiou and Chiou, 1990), the acquisition of this determinant is considered to be the result of antibiotic selection pressure. Although *C. difficile* is naturally resistant to aminoglycosides and the presence of this gene may not be necessarily correlated with aminoglycoside resistance in an anaerobic pathogen such as *C. difficile* (Kartalidis et al., 2021), the presence of this determinant is important for spreading to other pathogens. The Tn*6194*-like element was another transposon found discrepant in ST81 isolates, carrying oriented *erm*B gene and several insertion regions compared to the reference sequences and that in ST37. However, this did not appear to affect the susceptibility to MLS_*B*_ antibiotics as the resistance rates for clindamycin in both sequence types reached 100%. Notably, more than half of the genes in the insertion regions of these element in ST81 corresponded to the unique genes of the ST81 isolates. Generally, the ICEs in the ST81 isolates displayed significant homology with those in the ST37 isolates, whereas ST81 isolates exhibited potentially more dynamic in interspecies transmission.

Antimicrobial resistance in *C. difficile* could be the result of multiple mechanisms, including the presence of resistance genes carried by chromosomes and/or mobile genetic elements, the alteration of site-specific target genes, and so on (Peng et al., 2017). Although no obvious differences in resistance were found for tetracycline and MLS_*B*_ antibiotics conferred by ICEs, the remarkably higher levels of resistance to fluoroquinolones in the ST81 isolates in accordance with the alterations in *gyrA* and *gyrB* caught our attention (Cheng et al., 2020). Fluoroquinolone resistance resulting from the Thr82Ile mutation in *C. difficile* B1/NAP1/RT027/ST01 is considered to have facilitated the rapid expansion and dissemination of this strain during outbreaks in North America and Europe in the early 2000s (He et al., 2013). Moreover, the reduced susceptibility of ST81 isolates to fluoroquinolones suggests a potentially high risk of worldwide transmission. The amino acid substitution Thr82Ile in GyrA was essential to moxifloxacin resistance (Dridi et al., 2002), whereas the additional mutation of Asp426Val in GyrB led to increased MIC values for the ST81 isolates. Furthermore, phylogenetic analysis of the *gyrA* and *gyrB* mutations allowed the isolates to be grouped into two lineages, indicating that mutations in the *gyr* genes could serve as an indicator for ST81 isolates. Another finding concerning antimicrobial susceptibility is that the ST81 isolates exhibited reduced resistance rates toward rifampicin, along with lower levels of *rpoB* gene mutation compared with the ST37 isolates, which is consistent with other studies conducted in China (Cheng et al., 2020). Alteration of the housekeeping genes related to antibiotics was presumably the result of selective pressure *in vivo* during the development of the antibiotic-resistant phenotypes. Thus, the discrepancy of ST81 isolates with respect to rifampicin resistance may be associated with the clinical usage of this antibiotic, although this requires further confirmation.

Comparative genomic analysis revealed that a large number of the unique genes in the ST81 isolates were classified as being of unknown function, making it difficult to compare them with those in the ST37 isolates. Therefore, we explored non-synonymous SNPs in genes potentially related to the ability to survive in harsh environments and to transmit between hosts. SNPs accumulated in generation between *C. difficile* derivative strains are suggested to be responsible for the phenotypic differences (Janezic et al., 2018). Our *in vivo* experiments in mice demonstrated that the ST81 isolates were ultimately more competitive than the ST37 isolates. This robust colonization ability enabled ST81 to mediate widespread infection. This is consistent with the phenotypic profiling results, which included remarkable sporulation ability, enhanced epithelial cell adhesion, improved survival in macrophages, and slightly increased motility. Also, a substantial number of gene variants in SNPs between the ST81 and ST37 isolates were detected as relevant genes described above. The change of a single SNP in a single gene could not easily alter the entire transcriptome or the relevant phenotype (Collery et al., 2017). Thus, the results suggest that the altered properties of the ST81 isolates are likely to originate from a combination of the identified SNPs. The striking SNP differences observed in genes involved in sporulation [*cspA* and *cspBA* (Kevorkian and Shen, 2017)], biofilm formation [*pdcA* (Purcell et al., 2017)], oxidative stress [*treR* (Baker et al., 2018)], and multiple functions [*sinI* (Ciftci et al., 2019) and *rho* (Trzilova et al., 2020)] merit attention for further exploration of the pathogenesis of *C. difficile*.

However, our study has some limitations. First, our results may be regionally and temporally specific because the ST81 and ST37 isolates were collected from a single institution over a relatively short period of time. Second, only a limited number of representative strains were subjected to whole genome sequencing, which may have led to underestimation of the genomic heterogeneity within sequence types. Finally, the analyzed SNPs were mostly in functional genes reported previously, which may have resulted in important information for other genes being overlooked.

In conclusion, the results obtained in this study have afforded comprehensive insights into the genomic and phenotypic profiles of clinical ST81 and ST37 isolates. The phylogenetic and genomic features revealed that the epidemiologically important genotype ST81 is closely related to ST37, acting as a dynamic vehicle harboring similar ICEs with more diverse and insertion region. High levels of resistance to fluoroquinolones along with phenotypic advantages both *in vivo* and *in vitro* may contribute to the gradual clinical emergence of this strain. The additional *gyrB* mutation of Asp426Val is strongly related to the increased MIC values of the ST81 isolates with respect to fluoroquinolones. The diverse range of observed SNPs underlines the important roles of the genes responsible for the discussed phenotypes. However, additional investigations are required to shed further light on the molecular mechanisms underlying the evolution and transmission of the ST81 strain.

## Data Availability Statement

The dataset generated from this study has been deposited in the NCBI database under the BioProject number PRJNA752438. The genomes of *C. difficile* 630 (AM180355.1), *C. difficile* M68 (FN668375.1), *C. difficile* CD161 (CP029154.1), *C. difficile* CDT4 (CP029152.1), *C. difficile* CF5 (FN665652.1), *C. difficile* R20291 (FN545816.1), *C. difficile* DSM 29020 (CP012325.1), and *C. difficile* M120 (NC_017174.1) can be found in GenBank. All of the data in this study are available from the corresponding authors upon reasonable request.

## Ethics Statement

The studies involving human participants were reviewed and approved by The Ruijing Hospital Ethics Committee. Written informed consent for participation was not required for this study in accordance with the national legislation and the institutional requirements. The animal study was reviewed and approved by The Ruijing Hospital Ethics Committee.

## Author Contributions

TS, DD, and YP contributed to the conception and design of the study. DW, QN, CJ, and WC contributed to the collection of clinical isolates. TS, DW, YC, and WC contributed to the experiments. TS and DD performed the bioinformatics analysis and wrote the first draft of manuscript. All authors contributed to revising the manuscript and have approved the submitted version.

## Conflict of Interest

The authors declare that the research was conducted in the absence of any commercial or financial relationships that could be construed as a potential conflict of interest.

## Publisher’s Note

All claims expressed in this article are solely those of the authors and do not necessarily represent those of their affiliated organizations, or those of the publisher, the editors and the reviewers. Any product that may be evaluated in this article, or claim that may be made by its manufacturer, is not guaranteed or endorsed by the publisher.

## Footnotes

1
https://pubmlst.org/organisms/clostridioides-difficile

2
https://orthomcl.org/orthomcl/app/downloads/software

3
https://cloud.majorbio.com/

4
http://bio-bwa.sourceforge.net/

5
http://snpeff.sourceforge.net/SnpEff.html

6
https://github.com/davidemms/OrthoFinder/releases/tag/2.3.7

7
https://db-mml.sjtu.edu.cn/ICEberg2/index.php
