# Supplementary material for: Complete Genome Sequencing and Comparative Phenotypic Analysis Reveal the Discrepancy Between Clostridioides difficile ST81 and ST37 Isolates
Source: Front Microbiol. 2021 Dec 21;12:776892. doi: 10.3389/fmicb.2021.776892 (PMC8725731; doi:10.3389/fmicb.2021.776892)
Supplement: Supplementary file 1 [file Data_Sheet_1.PDF]

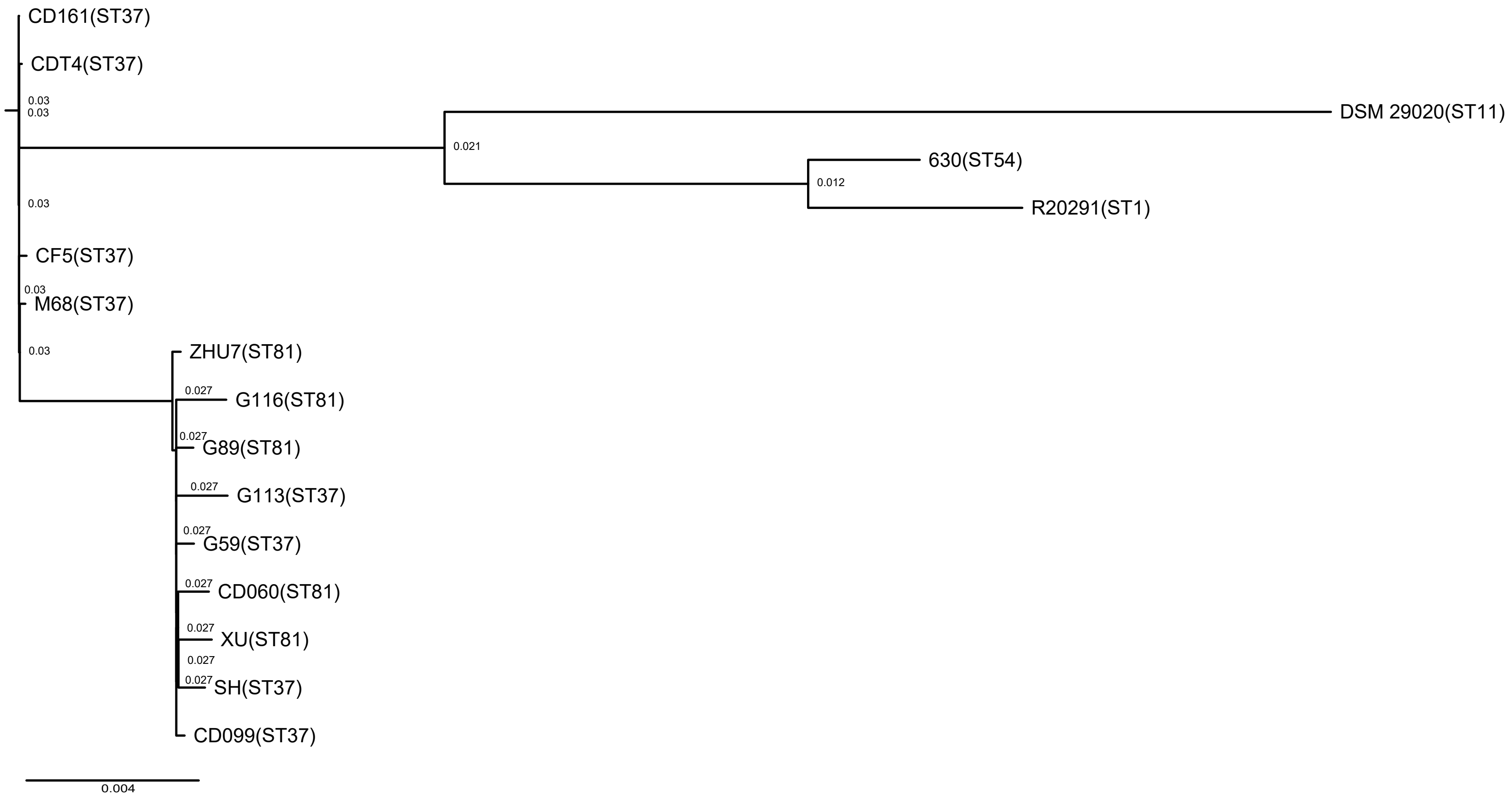

Supplementary Figure 1| Analysis of phylogenetic tree depicting the relationships of C. difficile isolates based on single copy orthologue sequences by draft genome sequencing. CD161, CDT4, CF5, M68, DSM 29020, 630 and R20291 were used as reference strains. The sequence types are labeled in parentheses.

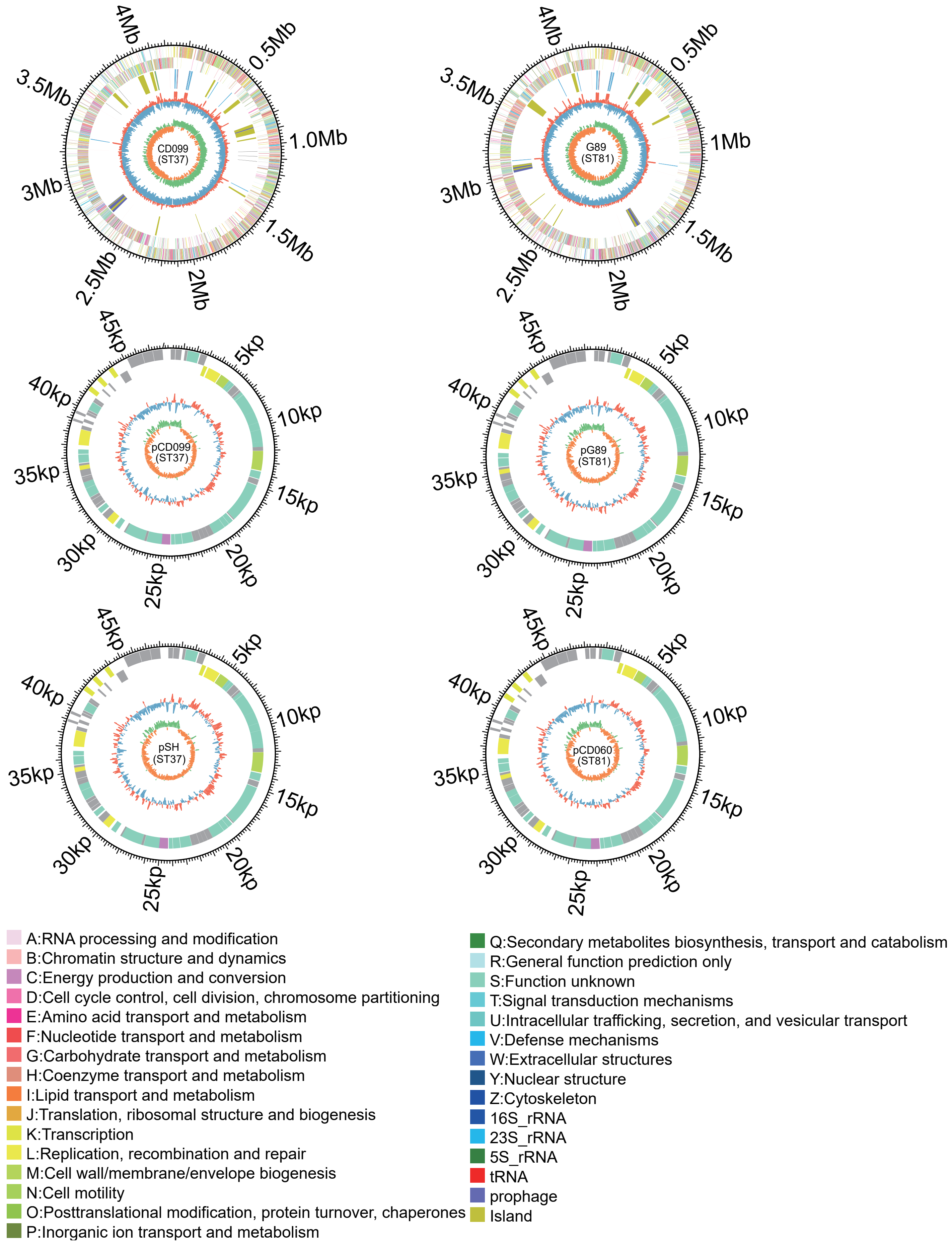

Supplementary Figure 2| Schematic diagrams of the complete chromosomal genomes and plasmid genomes. The circle diagram indicated as follows(from the outer layer inward): the genome size, the annotated COG genes on the forward strand, the annotated COG genes on the reverse strand, 16S rRNA, 23S rRNA, 5S rRNA, tRNA, prophage, island, GC content, and GC skew(G−C/G+C).

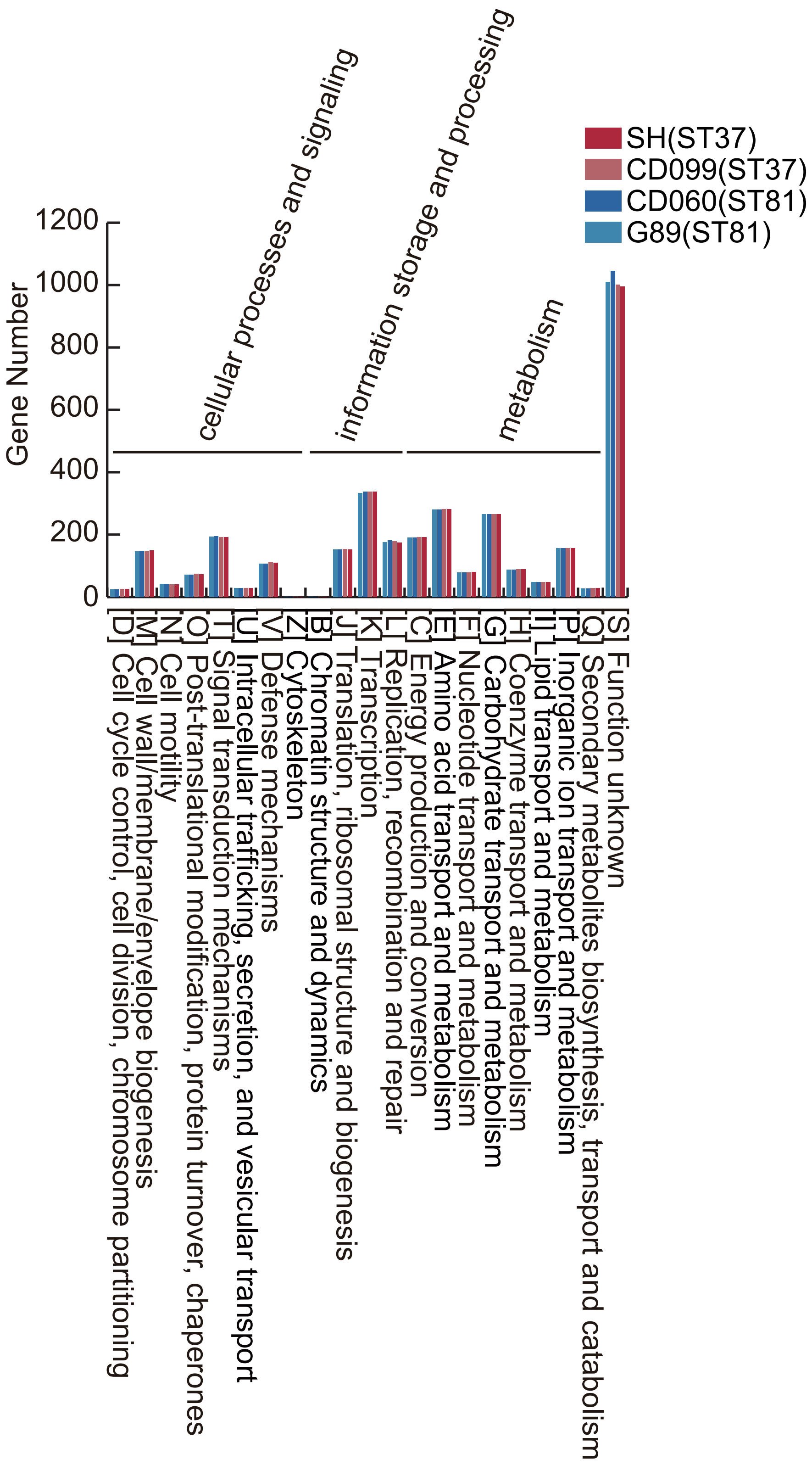

Supplementary Figure 3| COG annotation of total CDSs in the ST81 and ST37 isolates.

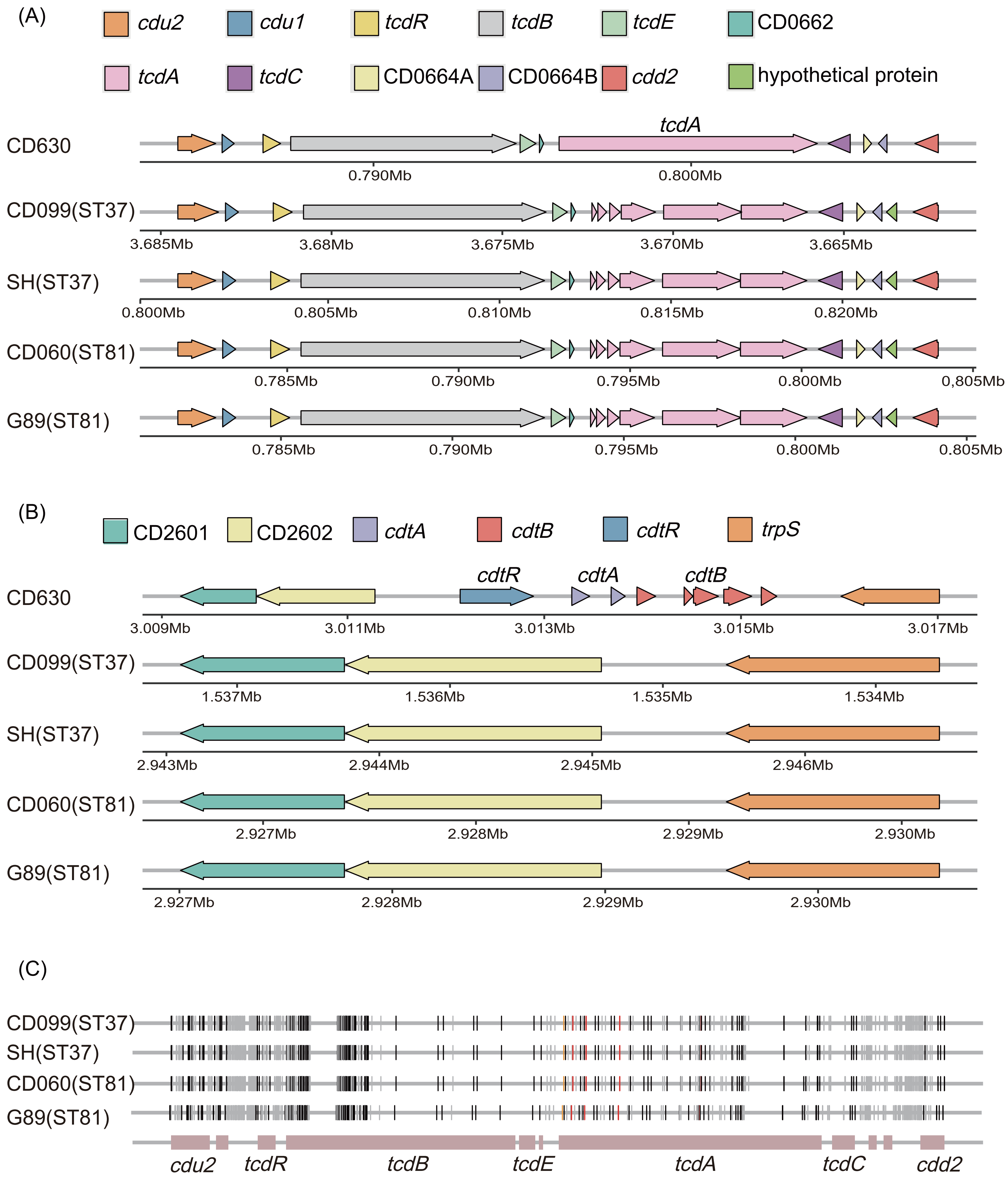

Supplementary Figure 4| Features of the PaLoc and CdtLoc regions in the ST81 and ST37 isolates. CD630 was used as a reference strain. (A) Schematic representation of the PaLoc region among these isolates. (B) Schematic representation of the CdtLoc region among these isolates. (C) Schematic representation of the SNPs in the PaLoc region among these isolates. Synonymous variants, missense variants, frameshift variants, and stop gain mutations are shown in gray, black, red, and orange, respectively.
