# Supplementary material for: Complete Genome Sequencing and Comparative Phenotypic Analysis Reveal the Discrepancy Between Clostridioides difficile ST81 and ST37 Isolates
Source: Front Microbiol. 2021 Dec 21;12:776892. doi: 10.3389/fmicb.2021.776892 (PMC8725731; doi:10.3389/fmicb.2021.776892)
Supplement: Supplementary file 2 [file Data_Sheet_2.PDF]

Supplementary Table 1| Genomic features of involved ST81 and ST37 isolates

Strain	Sequence Type	Genome Completion	Genome Size	GC%	CDSs	tRNAs	rRNAs	Plasmid	Prophages
630	54	complete	4290252	29.06	3776	87	11	1	2
CD060	81	complete	4338261	28.77	4009	89	35	1	3
G89	81	complete	4288937	28.76	3920	89	35	1	2
G116	81	draft	4272131	28.55	4002	22	3	-[a]	5
ZHU7	81	draft	4335376	28.37	4095	26	4	-	1
XU	81	draft	4387640	28.44	4158	21	5	-	2
M68	37	complete	4308325	28.90	3830	109	30	1	4
CD099	37	complete	4302821	28.80	3915	90	35	1	2
SH	37	complete	4287439	28.77	3898	90	35	1	3
G59	37	draft	4281610	28.63	3992	18	3	-	3
G113	37	draft	4264998	28.62	3934	18	3	-	3

[a] Not applicable

Supplementary Table 2| Integrative conjugative elements analyzed in this study

Reference					CD099(ST37)				SH(ST37)				CD060(ST81)				G89(ST81)			
ICE name	Referenced Isolates	ORF	putative conjugative machinery	Specific gene	matched ORF	putative conjugative machinery	Integrase	Start-end	matched ORF	putative conjugative machinery	Integrase	Start-end	matched ORF	putative conjugative machinery	Integrase	Start-end	matched ORF	putative conjugative machinery	Integrase	Start-end
CTn1	630	33	T4SS, relaxase	int	9	T4SS [a]	- [b]	gene0663-gene0637	9	T4SS	-	gene3564-gene3588	11	T4SS	int	gene3450-gene3473	11	T4SS	int	gene3366-gene3388
CTn2	630	35	T4SS, relaxase	integrase	14	T4SS, relaxase	-	gene3729-gene3688	14	T4SS, relaxase	-	gene0515-gene0557	15	T4SS, relaxase	site-specific recombinase	gene0491-gene0542	15	T4SS, relaxase	site-specific recombinase	gene0491-gene0543
Tn5397	630	24	T4SS, relaxase	tetM, tndX	9	T4SS, relaxase	-	gene3787-gene3778	9	T4SS, relaxase	-	gene0458-gene0467	9	T4SS, relaxase	-	gene3869-gene3879	9	T4SS, relaxase	-	gene3782-gene3791
CTn5	630	38	T4SS, relaxase	integrase	31	T4SS, relaxase	integrase	gene3732-gene3698	31	T4SS, relaxase	integrase	gene0513-gene0547	32	T4SS, relaxase	integrase, site-specific recombinase	gene0489-gene0533	32	T4SS, relaxase	integrase, site-specific recombinase	gene0489-gene0533
CTn6	630	24	T4SS, relaxase	integrase	1	-	-	gene0403	2	-	-	gene0404-gene0405	-	-	-	-	-	-	-	-
CTn7	630	30	T4SS, relaxase	integrase	11	T4SS, relaxase	-	gene0663-gene0649	10	T4SS, relaxase	-	gene3564-gene3578	11	T4SS, relaxase	-	gene3450-gene3463	10	T4SS, relaxase	-	gene3366-gene3378
Tn916	E.faecalis DS16	24	T4SS, relaxase	tetM, int-Tn	10	T4SS, relaxase	-	gene3387-gene3377	10	T4SS, relaxase	-	gene0458-gene0468	10	T4SS, relaxase	-	gene3869-gene3860	10	T4SS, relaxase	-	gene3782-gene3792
Tn6103	R20291	71	T4SS, relaxase	recombinase	18	T4SS, relaxase	-	gene3733-gene3697	18	T4SS, relaxase	-	gene0512-gene0548	19	T4SS, relaxase	site-specific recombinase	gene0488-gene0534	19	T4SS, relaxase	site-specific recombinase	gene0488-gene0534
Tn6194-like	CII7	35	T4SS, relaxase	emrB, int	29	T4SS, relaxase	int	gene0664-gene0636	27	T4SS, relaxase	int	gene3563-gene3589	13	T4SS, relaxase	-	gene3449-gene3467	14	T4SS, relaxase	-	gene3365-gene3383

[a] type IV secretion system, [b] Not applicable

Supplementary Table 3| Summary of SNPs and Indels from ST81 and ST37 isolates against reference genome

Regions	Categories	CD099(ST37)	SH(ST37)	CD060(ST81)	G89(ST81)
exon	synonymous variant	28,440	28,461	25194	25,170
	missense variant	11,992	12,000	10,837	10,815
	frameshift variant	114	113	110	111
	inframe deletion	35	34	36	35
	inframe insertion	37	36	38	39
	start lost	45	45	40	40
	stop lost	46	46	45	44
	stop gained	36	38	38	37
others[a]	-[b]	10,708	10744	9814	9818

[a] including upstream, downstream, intergenic, intron and splice site. [b] Not applicable

Supplementary Table 4| Different SNPs in functional genes of ST81 and ST37 isolates

Name	GeneID	Gene Function	Description	Amino Acid Mutations			
				CD099(ST37)	SH(ST37)	CD060(ST81)	G89(ST81)
htrA	CD630_32840	ahension, sporulation	Serine protease, HrtA family	N261T	N261T	-[a]	-
sinI	CD630_22150	sporulation, motility	Transcriptional regulator, HTH-type	N24S, N53S, I54V, V61I	N24S, N53S, I54V, V61I	-	-
rho	CD630_34870	sporulation, motility	Transcription termination factor Rho	H109N, K69R	H109N, K69R	-	-
sigK	CD630_12300	sporulation	Sigma K	L128F, I127T	L128F, I127T	L128F, I127T, T117I	L128F, I127T, T117I
gerS	CD630_34640	sporulation	putative lipoprotein	-	-	G94D	G94D
alr2	CD630_34630	sporulation	Alanine racemase 2	G276D, D90E	G276D, D90E	E145K	E145K
cspC	CD630_22460	sporulation	Subtilisin-like serine germination related protease	T477A, N394G, V320I, S290N, S250R, I184V, V179A, T121S, L39I, R24K, D12N	T477A, N394G, V320I, S290N, S250R, I184V, V179A, T121S, L39I, R24K, D12N	D415S	D415S
cspBA	CD630_22470	sporulation	Subtilisin-like serine germination related protease	I917V, D884E, D794G, A766S, S629N, S622F, R610N, S608G, I592V, M540I, I47T	I917V, D884E, D794G, A766S, S629N, S622F, R610N, S608G, I592V, M540I, I47T	S629N, M540I	S629N, M540I
CD1231	CD630_12310	sporulation	putative site-specific recombinase	D504E, N478D, I366L, A363S, E15D	D504E, N478D, I366L, A363S, E15D	N478D, V233A	N478D, V233A
bclA1	CD630_03320	sporulation	putative exosporium glycoprotein	490GNT491, V40I, T466M, E480S, A486V, T526M, A531P, V534A, P540A, A543P, T587A, V643I	V40I, T526M, A531P, V534A, P540A, A543P, T587A, V643I	446deletion450, 490GNT491, V40I, A450P, T466M, E480S, A486V, T526M, A531P, V534A, P540A, A543P, T587A, V643I	446deletion450, 490GNT491, V40I, A450P, T466M, E480S, A486V, T526M, A531P, V534A, P540A, A543P, T587A, V643I
CD1492	CD630_14920	sporulation	Two-component sensor histidine kinase,sporulation-associated spo0A	T49L, I150V, I154V, H174R, L207V, N395S, V432A, E457K, V874I, S879N	T49L, I150V, I154V, H174R, L207V, N395S, V432A, E457K, V874I, S879N	N830K, S879N, I882V	N830K, S879N, I882V
cotB	CD630_15110	sporulation	spore coat protein	N189S, N112D, V83A, T58A	N189S, N112D, V83A, T58A	-	-

pdcA	CD630_15150	biofilm formation	diguanylate kinase signaling protein	H4R, K56Q, V90A, S119I, P123A, H130N, N135T, K196E, V200I, K203E, D309N, N371D, E471Q, I475M, C489Y, Y528N, E550N, M579L, F584S	H4R, K56Q, V90A, S119I, P123A, H130N, N135T, K196E, V200I, K203E, D309N, N371D, E471Q, I475M, C489Y, Y528N, E550N, M579L, F584S	I336V	I336V
CD2215	CD630_22150	biofilm formation	Transcriptional regulator, HTH-type	N24S, N53S, I54V, V61I	N24S, N53S, I54V, V61I	-	-
treR	CD630_30900	stress reaction	Transcriptional regulator, GntR family	T54A, N104H, D154N, C171S, 240frameshift	T54A, N104H, D154N, C171S, 240frameshift	N104H	N104H
gyrB	CD630_00060	antimircrobial suceptibility	DNA gyrase subunit B	S366A	S366A, D465N	S366A, D426V	S366A, D426V
tetM	CD630_05080	antimircrobial suceptibility	Tetracycline resistance protein Tn5397	T26N, A31T, T33L, T38K, T45N, F47L, A59G, V60I, K66E, D67N, I68T, R102K, N117T, M150A, I153V, H161Y, M167T, S168N, C169F, P172S, V177T, Y184D, V216L, E228D, Q229N, Y243H, K245G, K246P, S259T, E260K, E261K, V268I, G272S, I274V, I283V, I291V, K207R, E337K, R338K, L343H, Q356E, Q376R, T399I, C400S, V411I, K412E, I413L, K415E, K427N, A450S, V458M, N591S, Y611H, S617P	T26N, A31T, T33L, T38K, T45N, F47L, A59G, V60I, K66E, D67N, I68T, R102K, N117T, M150A, I153V, H161Y, M167T, S168N, C169F, P172S, V177T, Y184D, Y214F, V216L, E228D, Q229N, Y243H, K245G, K246P, S259T, E260K, E261K, V268I, G272S, I274V, I283V, I291V, K207R, E337K, R338K, L343H, Q356E, Q376R, T399I, C400S, V411I, K412E, I413L, K415E, K427N, A450S, V458M, N591S, Y611H, S617P	T26N, A31T, T33L, T38K, T45N, F47L, A59G, V60I, K66E, D67N, I68T, R102K, N117T, M150A, I153V, H161Y, M167T, S168N, C169F, P172S, V177T, Y184D, E228D, Q229N, Y243H, K245G, K246P, S259T, E260K, E261K, V268I, G272S, I274V, I283V, I291V, K207R, E337K, R338K, L343H, Q356E, Q376R, T399I, C400S, V411I, K412E, I413L, K415E, K427N, A450S, V458M, N591S, Y611H, S617P	T26N, A31T, T33L, T38K, T45N, F47L, A59G, V60I, K66E, D67N, I68T, R102K, N117T, M150A, I153V, H161Y, M167T, S168N, C169F, P172S, V177T, Y184D, V216L, E228D, Q229N, Y243H, K245G, K246P, S259T, E260K, E261K, V268I, G272S, I274V, I283V, I291V, K207R, E337K, R338K, L343H, Q356E, Q376R, T399I, C400S, V411I, K412E, I413L, K415E, K427N, A450S, V458M, N591S, Y611H, S617P

[a] No mutation identified

Supplementary Table 5| Antimicrobial susceptibility of isolates in this study

Antimicrobial agents	ST37(n=16)				ST81(n=34)				CD630
	MIC50(mg/L)	MIC90(mg/L)	MIC range	Resistant(%)	MIC50(mg/L)	MIC90(mg/L)	MIC range	Resistant(%)	MIC(mg/L)
Penicillin	2	4	1-4	56.25%	2	4	0.5-8	67.65%	4
Rifampicin	<1	>64	<1->64	43.75%	<1	<1	<1->64	2.94%	<1
Clindamycin	>128	>128	>128	100%	>128	>128	16->128	100%	>128
Tetracycline	16	32	0.25-32	81.25%	32	32	1-32	88.24%	64
Cefotaxime	>128	>128	>128	100%	>128	>128	64->128	100%	128
Cefoxitin	>128	>128	>128	100%	>128	>128	64->128	100%	>128
Moxifloxacin	64	64	2-64	62.50%	>128	>128	2->128	94.12%	2
Metronidazole	<0.125	<0.125	<0.125-0.25	0%	<0.125	0.5	<0.125-0.5	0%	<0.125
Vancomycin	0.5	0.5	0.5	0%	0.5	1	0.25-1	0%	0.5
